# Sepsis and the Liver

**DOI:** 10.3390/diseases13120388

**Published:** 2025-11-28

**Authors:** Eleni V. Geladari, Anastasia-Amalia C. Kalergi, Apostolos A. Evangelopoulos, Vasileios A. Sevastianos

**Affiliations:** 1 3rd Department of Internal Medicine & Liver Unit, “Evangelismos” General Hospital, 45-47 Ipsilantou Str, 106 76 Athens, Greece or elgeladari@gmail.com (E.V.G.); gpath2@evaggelismos-hosp.gr (A.-A.C.K.); 2 Medical School, National and Kapodistrian University of Athens, 115 27 Athens, Greece; apostolos.evangelopoulos.nak@gmail.com

**Keywords:** sepsis-associated liver injury (SALI), inflammatory cascade, mitochondrial dysfunction, gut–liver axis, biomarkers

## Abstract

Background/Objectives: Sepsis-associated liver injury (SALI) is a critical and often early complication of sepsis, defined by distinct hyper-inflammatory and immunosuppressive phases that shape patient phenotypes. Methods: Characterizing these phases establishes a foundation for immunomodulation strategies tailored to individual immune responses, as discussed subsequently. Results: The initial inflammatory response activates pathways such as NF-κB and the NLRP3 inflammasome, leading to a cytokine storm that damages hepatocytes and is frequently associated with higher SOFA scores and a higher risk of 28-day mortality. Kupffer cells and infiltrating neutrophils exacerbate hepatic injury by releasing proinflammatory cytokines and reactive oxygen species, thereby causing cellular damage and prolonging ICU stays. During the subsequent immunosuppressive phase, impaired infection control and tissue repair can result in recurrent hospital-acquired infections and a poorer prognosis. Concurrently, hepatocytes undergo significant metabolic disturbances, notably impaired fatty acid oxidation due to downregulation of transcription factors such as PPARα and HNF4α. This metabolic alteration corresponds with worsening liver function tests, which may reflect the severity of liver failure in clinical practice. Mitochondrial dysfunction, driven by oxidative stress and defective autophagic quality control, impairs cellular energy production and induces hepatocyte death, which is closely linked to declining liver function and increased mortality. The gut-liver axis plays a central role in SALI pathogenesis, as sepsis-induced gut dysbiosis and increased intestinal permeability allow bacterial products, including lipopolysaccharides, to enter the portal circulation and further inflame the liver. This process is associated with sepsis-related liver failure and greater reliance on vasopressor support. Protective microbial metabolites, such as indole-3-propionic acid (IPA), decrease significantly during sepsis, removing key anti-inflammatory signals and potentially prolonging recovery. Clinically, SALI most commonly presents as septic cholestasis with elevated bilirubin and mild transaminase changes, although conventional liver function tests are insufficiently sensitive for early detection. Novel biomarkers, including protein panels and non-coding RNAs, as well as dynamic liver function tests such as LiMAx (currently in phase II diagnostics) and ICG-PDR, offer promise for improved diagnosis and prognostication. Specifying the developmental stage of these biomarkers, such as identifying LiMAx as phase II, informs investment priorities and translational readiness. Current management is primarily supportive, emphasizing infection control and organ support. Investigational therapies include immunomodulation tailored to immune phenotypes, metabolic and mitochondrial-targeted agents such as pemafibrate and dichloroacetate, and interventions to restore gut microbiota balance, including probiotics and fecal microbiota transplantation. However, translational challenges remain due to limitations of animal models and patient heterogeneity. Conclusion: Future research should focus on developing representative models, validating biomarkers, and conducting clinical trials to enable personalized therapies that modulate inflammation, restore metabolism, and repair the gut-liver axis, with the goal of improving outcomes in SALI.

## 1. Introduction

Sepsis represents a leading cause of mortality and critical illness worldwide [1]. Rather than a simple infection, sepsis is characterized by a dysregulated host response that results in inflammation, immune suppression, and organ failure. The liver serves both as a primary defense against infection and as a highly vulnerable organ during sepsis [2,3]. Historically, sepsis-associated liver injury (SALI) was regarded as a late-stage complication within multiple organ dysfunction syndrome (MODS). However, recent evidence identifies SALI as an early and independent predictor of mortality, underscoring its clinical importance [4,5].

The liver is essential for immune defense, metabolic regulation, and detoxification. Hepatic dysfunction impairs pathogen and toxin clearance, diminishes acute-phase protein synthesis, and disrupts inflammation regulation [6,7]. These problems hinder infection control, worsen sepsis, and lead to organ failure. Each year, sepsis affects millions of people. Liver dysfunction in sepsis is linked with high mortality, exceeding 60% in intensive care [1,8,9]. Understanding the mechanisms behind SALI is critical for developing new therapies and lowering death rates [10,11]. The liver plays a central role in immune defense, metabolic regulation, and detoxification. Hepatic dysfunction compromises the clearance of pathogens and toxins, reduces acute-phase protein synthesis, and disrupts the regulation of inflammation [6,7]. These impairments impede infection control, exacerbate sepsis, and contribute to organ failure. Sepsis affects millions of individuals annually, and liver dysfunction in this context is associated with mortality rates exceeding 60% in intensive care settings [1,8,9]. Elucidating the mechanisms underlying SALI is essential for developing novel therapies and reducing mortality [10,11].

Despite substantial research efforts, the molecular and cellular mechanisms underlying SALI remain poorly understood. Immune and inflammatory dysregulation, metabolic and mitochondrial disturbances, and disruptions of the gut–liver axis have been identified as principal contributors. These interconnected processes drive liver injury, cholestasis, and impaired hepatic clearance, thereby exacerbating sepsis [5,10,12,13,14,15,16,17]. The absence of mechanistic clarity impedes the development of targeted therapies and early diagnostic tools, perpetuating high morbidity and mortality rates.

This review provides a comprehensive summary of current evidence regarding liver dysfunction in sepsis, with particular emphasis on inflammatory pathways, metabolic alterations, and the role of the gut microbiota. By integrating molecular, cellular, and clinical perspectives, the review aims to clarify ongoing controversies, identify knowledge gaps, and propose directions for future research. Key debates include the timing and impact of immune suppression, inconsistent findings regarding cytokines such as TNF-alpha and interleukin-6, and the therapeutic potential of targeting mitochondrial dysfunction. Additional unresolved issues involve the diagnostic utility of liver function tests and strategies to restore gut microbiota balance. Addressing these challenges is essential for advancing personalized therapies and early intervention. This review is intended to guide innovation and facilitate the translation of research findings into improved clinical outcomes [7,10].

## 2. Epidemiology

Sepsis-induced liver injury frequently occurs in critically ill and cirrhotic patients, significantly worsening prognosis [8]. Sepsis affects millions of individuals worldwide each year, and liver injury further worsens the prognosis [3,8,18]. While the liver frequently demonstrates a capacity for recovery, the development of liver failure is associated with a marked increase in mortality risk [3].

Liver dysfunction develops in approximately 34% to 46% of patients with sepsis [13,19]. This prevalence underscores the liver’s particular susceptibility compared to other sepsis-related complications, such as renal dysfunction, which occurs in 23% to 35% of cases, and pulmonary dysfunction, which affects 30% to 40%. The relatively higher incidence of liver dysfunction emphasizes its pivotal role in the progression and outcomes of sepsis.

Within intensive care units (ICUs), sepsis rates can reach 20%, and liver injury constitutes a common form of organ failure among these patients [19].

Sepsis occurs with greater frequency and severity in patients with liver cirrhosis. This population faces a significantly elevated risk of developing sepsis and subsequent organ failure compared to individuals without cirrhosis [3,20].

Mortality rates for sepsis patients with liver dysfunction range from 54% to 68%, exceeding those observed with dysfunction of other organs, such as the lungs [3,18].

Liver injury is an independent predictor of poor prognosis and increased mortality in sepsis [3,19]. Patients with pre-existing liver conditions, including cirrhosis, trauma, or alcohol dependence, are at particularly high risk for sepsis. These underlying conditions further elevate the likelihood of multiple organ failure and sepsis-related mortality [3,20].

Liver dysfunction often appears early in the course of sepsis, sometimes within hours, and may signal the onset of multiple organ dysfunction [5,19]. Early clinical signs include mild jaundice, subtle confusion, or abrupt changes in coagulation parameters. Laboratory indicators important for early detection are slight increases in ALT, AST, and bilirubin levels. The rapid onset of hepatocyte injury is primarily attributed to cytokine-mediated microcirculatory collapse, highlighting the need for clinicians to anticipate and monitor for early deterioration. The early onset of hepatic dysfunction is linked to worse clinical outcomes and higher mortality rates [20].

Older adults, individuals with liver cirrhosis, alcohol dependence, or comorbidities such as diabetes or cardiac dysfunction are at increased risk for sepsis-associated liver injury relative to the general sepsis population [18,20]. To efficiently identify these high-risk individuals upon admission, a simple checklist can be used to guide screening decisions: “screen liver enzymes such as AST, ALT, and bilirubin on admission if the patient is 65 years or older, has a history of liver cirrhosis, alcohol use disorder, diabetes, or cardiac issues”. Implementing practical screening cues at the bedside may empower medical teams to promptly identify potential liver dysfunction, thereby improving patient outcomes.

Gender-based differences have been documented: certain studies indicate that females with cirrhosis are more prone to infectious complications, while other evidence suggests that males may experience higher mortality from acute liver injury during sepsis [5,20].

## 3. The Pathophysiological Triumvirate

**Figure 1:**
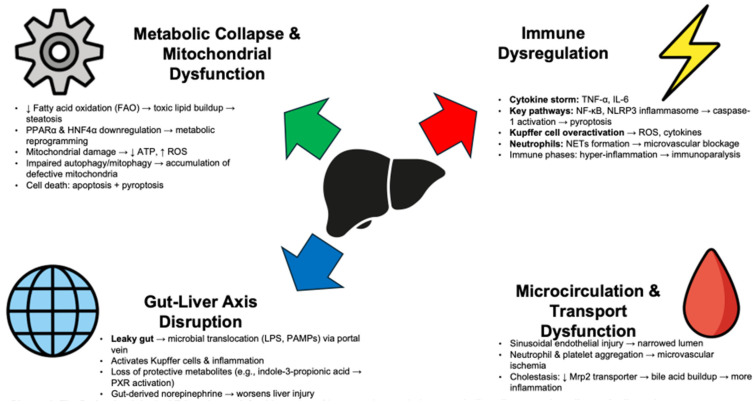
The Pathophysiological Triumvirate—A fatal interplay of immune dysregulation, metabolic collapse, and gut–liver axis disruption.

SALI develops through complex, interconnected events. Reduced hepatic blood flow and ischemia alone do not cause SALI. Instead, inflammation, metabolic changes, and organ interactions drive its onset. Immune dysregulation, metabolic collapse, and disruption of the gut–liver axis together cause liver dysfunction in sepsis (Figure 1 and Table 1).

### 3.1. The Inflammatory Cascade and Immune Dysregulation

The hallmark of sepsis is a dysregulated immune response, and the liver, with its rich population of resident immune cells, is at the epicenter of this storm. The initial response to pathogens involves the release of a torrent of proinflammatory cytokines (proteins that signal immune responses), often termed a “cytokine storm”, with tumor necrosis factor-alpha (TNF-α) and interleukin-6 (IL-6) being primary instigators. These mediators trigger widespread hepatocellular (liver cell) dysfunction, often occurring early in sepsis and independent of significant perfusion deficits, highlighting a direct inflammatory cause [21,22].

This cytokine surge activates canonical intracellular inflammatory signaling pathways [7]. The nuclear factor-kappa B (NF-κB) pathway, a master regulator of inflammation, consistently drives SALI. Its activation triggers the transcription of many genes encoding cytokines, chemokines, and adhesion molecules, thereby perpetuating inflammation [23,24,25]. At the same time, the NOD-, LRR-, and pyrin domain-containing protein 3 (NLRP3) inflammasome is activated in hepatocytes and immune cells. This multi-protein complex processes pro-caspase-1 into its active form, which then cleaves pro-IL-1β and pro-IL-18 into their mature, highly proinflammatory forms [26]. Furthermore, caspase-1 activation can trigger a lytic, proinflammatoryform of cell death called pyroptosis, which directly causes hepatocellular damage [13,23,27].

The liver’s distinctive immune microenvironment influences this process. Kupffer cells, which constitute the largest group of resident macrophages, act as the first line of defense by clearing pathogens and cellular debris from portal blood. However, in sepsis, they become overactive and inflict liver injury by producing high levels of cytokines and reactive oxygen species (ROS) [28,29]. Neutrophils also flood the liver, where they intensify injury by releasing cytotoxic granules and forming neutrophil extracellular traps (NETs), which can obstruct sinusoids and drive microvascular thrombosis [16,30].

This initial hyper-inflammatory phase often leads to immune suppression, or “immunoparalysis”, characterized by altered macrophage polarization and T-cell exhaustion. This shift hampers bacterial clearance, raises the risk of secondary infections, and complicates tissue repair, making the clinical course more difficult [31,32,33]. The complement system, a crucial part of innate immunity, also activates during sepsis and heightens hepatic inflammation and injury through direct cell lysis and opsonization [34,35]. This complex and dynamic immune dysregulation, involving both hyper-inflammation and subsequent suppression, drives the liver’s failure to maintain homeostasis during sepsis.

### 3.2. Metabolic Reprogramming and Mitochondrial Dysfunction

While inflammation sets the stage, the metabolic collapse within the hepatocyte is what ultimately cripples liver function. Sepsis induces a profound state of metabolic reprogramming, shifting the liver from a state of anabolic homeostasis to catabolic distress. A key feature of this dysregulation is the impairment of fatty acid oxidation (FAO). Under normal conditions, the liver relies on FAO for energy. In sepsis, this process is severely disrupted, leading to the accumulation of toxic lipid intermediates and hepatic steatosis (lipotoxicity), which further damages hepatocytes [10,15].

This metabolic failure is orchestrated at the transcriptional level by the dysfunction of key nuclear receptors. Peroxisome proliferator-activated receptor alpha (PPARα), the master regulator of hepatic lipid metabolism and FAO, is significantly downregulated during sepsis [15,36,37]. Similarly, hepatocyte nuclear factor 4-alpha (HNF4α), another critical regulator of hepatic metabolism, has been shown to be dysfunctional in polymicrobial sepsis, contributing to metabolic reprogramming and mortality [37]. The disruption of these transcriptional networks represents a core mechanism of metabolic collapse in SALI.

At the heart of this metabolic crisis lies the mitochondrion. Mitochondria are not only the powerhouses of the cell but are also central hubs for signaling, ROS production, and apoptosis. In sepsis, mitochondria are subjected to a multi-pronged assault. Oxidative stress, resulting from an imbalance between ROS production by immune cells and dysfunctional mitochondria and the cell’s antioxidant capacity, leads to widespread damage to mitochondrial DNA, proteins, and lipids [38,39]. This damage impairs the electron transport chain, leading to a catastrophic drop in ATP production and an energy crisis within the hepatocyte [17,40].

To cope with this damage, cells employ quality control mechanisms, primarily autophagy and mitophagy. Autophagy is a cellular recycling process that degrades and removes damaged organelles and proteins, while mitophagy is the specific autophagic removal of dysfunctional mitochondria. These processes are crucial adaptive responses that limit ROS production and maintain a healthy mitochondrial pool [41]. However, in severe sepsis, these pathways can become overwhelmed or impaired. Evidence suggests that TLR4 and TLR9 signaling are essential for sepsis-induced mitochondrial biogenesis, a process dependent on functional autophagy [42]. When these quality control mechanisms fail, damaged mitochondria accumulate, amplifying oxidative stress, depleting cellular energy, and ultimately triggering cell death pathways, including apoptosis and pyroptosis [41,43]. This convergence of metabolic reprogramming and mitochondrial collapse is a lethal blow to hepatic function.

### 3.3. The Gut–Liver Axis: A Critical Crosstalk

The gut–liver axis describes the intimate, bidirectional relationship between the gut and the liver, linked anatomically by the portal vein. In recent years, disruption of this axis has emerged as a pivotal mechanism in the pathogenesis of SALI [7,16]. The gut contains a vast and complex ecosystem of microorganisms—the gut microbiota—which plays a crucial role in maintaining host health. In sepsis, this delicate balance is shattered [8].

Sepsis is frequently associated with gut dysbiosis, a profound alteration in the composition and function of the gut microbiota. This is often accompanied by a breakdown of the intestinal barrier integrity, a condition colloquially known as “leaky gut” [8,12]. This increased permeability allows the translocation of viable bacteria and, more commonly, pathogen-associated molecular patterns (PAMPs), such as lipopolysaccharide (LPS), from the gut lumen directly into the portal circulation [13,44].

The liver, as the first organ to receive portal blood, is immediately inundated with these proinflammatory stimuli. Translocated PAMPs are potent activators of Kupffer cells and other hepatic immune cells, triggering and sustaining the inflammatory cascades described earlier [16,45]. This constant inflammatory pressure from the gut exacerbates direct hepatic injury and contributes to the systemic inflammatory response. Furthermore, some studies suggest that gut-derived norepinephrine released during sepsis can also contribute to hepatocellular dysfunction by activating Kupffer cells [46].

Beyond PAMPs, the metabolic output of the gut microbiota is also critical. Gut microbes produce a vast array of metabolites that can influence host physiology. For instance, indole-3-propionic acid (IPA), a metabolite produced by certain gut bacteria, has been shown to be protective against SALI. IPA can activate the pregnane X receptor (PXR), a nuclear receptor that regulates detoxification and inflammatory pathways, thereby alleviating liver injury [24]. A decrease in IPA-producing bacteria during sepsis could therefore remove a crucial protective mechanism. The disruption of the gut–liver axis thus represents a powerful “second hit” in SALI, amplifying the initial inflammatory insult and perpetuating a cycle of hepatic and systemic injury.

### 3.4. Hepatic Microcirculation and Transport Dysfunction

While systemic hypotension and shock can certainly cause ischemic liver injury, more subtle microcirculatory disturbances are now recognized as key contributors to SALI even in hemodynamically stable patients. Sepsis induces sinusoidal endothelial cell injury and swelling, leading to a narrowing of the sinusoidal lumen and impaired microvascular blood flow [47,48]. The activation of neutrophils and platelets leads to their aggregation and adhesion within the sinusoids, further disrupting microcirculation and promoting localized ischemia and inflammation [30,49].

This microcirculatory failure has profound consequences for the liver’s transport functions. One of the most common manifestations of SALI is cholestasis, the impairment of bile flow. This is not simply a mechanical obstruction but a functional failure of hepatocyte transporters. For example, studies have shown that sepsis-induced PI3Kγ signaling reduces the surface expression of the multidrug resistance-associated protein 2 (Mrp2), a key canalicular transporter responsible for bile acid excretion [50]. The resulting intrahepatic accumulation of cytotoxic bile acids causes further hepatocellular damage and worsens inflammation [12,14,51]. This combination of microvascular dysfunction and transporter failure creates a state of “intrahepatic gridlock”, trapping toxic substances within the liver and crippling its excretory capacity.

## 4. Clinical Manifestations and Diagnostic Challenges

The clinical presentation of SALI is highly variable, ranging from asymptomatic, mild elevations in liver enzymes to severe cholestatic jaundice, coagulopathy, and, in rare cases, fulminant hepatic failure. The most common pattern is “septic cholestasis”, characterized by elevated bilirubin levels with only modest increases in aminotransferases [11]. However, diagnosing SALI and predicting its severity using conventional laboratory tests is challenging (Table 2).

Standard liver function tests (LFTs), such as serum levels of alanine aminotransferase (ALT), aspartate aminotransferase (AST), and bilirubin, are often used to monitor hepatic function. Unfortunately, these markers are frequently late indicators of injury and lack specificity for sepsis. Elevations can be delayed and may not correlate well with the degree of functional impairment, limiting their utility for early diagnosis and prognostication. This diagnostic lag is a significant clinical problem, as early intervention is critical for improving outcomes.

Recognizing these limitations, research has focused on identifying novel biomarkers to enable earlier and more sensitive detection of SALI. Several promising candidates have emerged from preclinical and clinical studies:

Some molecules are not just markers but active participants in the injury process. Procalcitonin, a well-known sepsis biomarker, has been shown to directly impair hepatocyte viability in vitro. Its potential bedside trigger could be its elevation in serum levels, prompting early escalation of antibiotic therapy to combat a suspected infection worsened by liver dysfunction [52]. Similarly, CAAP48 (circulating alpha-1-antitrypsin protein fragment) has been identified as a novel biomarker that induces hepatic dysfunction in a liver-on-a-chip model, suggesting it could serve as a potential indicator of sepsis severity and the need for intensive monitoring [53].

Studies have identified panels of proteins that may offer better diagnostic accuracy. A panel including arginase-1 (ARG1), malate dehydrogenase 1 (MDH1), glutathione S-transferase alpha (GSTα), 5′-nucleotidase (5-NT), and succinate dehydrogenase (SDH) has shown promise for the early detection of hepatic dysfunction in critically ill sepsis patients. These panels could guide decisions on the need for liver support interventions, depending on the degree of marker elevation [54].

Non-coding RNAs (ncRNAs), such as microRNAs and long non-coding RNAs, are emerging as key regulators of gene expression in SALI. They modulate oxidative stress, cytokine responses, and cell death pathways, and their presence in circulation makes them attractive candidates for liquid biopsy-based diagnostics. They might be used to decide on the timing of immunomodulatory therapies [55].

Beyond static biomarkers, dynamic liver function tests that measure the liver’s actual metabolic capacity are gaining traction. The Lidocaine-Metabolite (LiMAx) test, which measures the liver’s capacity to metabolize ^13^C-methacetin, and the Indocyanine Green Plasma Disappearance Rate (ICG-PDR), have been shown to outperform traditional LFTs and scoring systems in predicting mortality and liver dysfunction in septic patients [56]. Implementation of these tests could influence crucial treatment decisions regarding organ support strategies.

## 5. Therapeutic Horizons: From Bench to Bedside

Despite a deepening understanding of its pathophysiology, the management of SALI remains largely supportive, focused on treating the underlying infection, maintaining hemodynamic stability, and providing organ support [13,57]. There are no approved therapies that specifically target the mechanisms of liver injury in sepsis, reflecting a significant “translational chasm” between promising findings in preclinical models and successful clinical application [58]. Nonetheless, ongoing research is exploring several therapeutic avenues that target the core pillars of SALI pathophysiology (Table 3).

Among the most advanced translational candidates are therapies targeting mitochondrial dysfunction and metabolic modulation, with compounds such as dichloroacetate and pemafibrate showing significant promise in preclinical studies and advancing towards clinical trials [59]. Additionally, novel diagnostics, such as the Indocyanine Green Plasma Disappearance Rate (ICG-PDR), are advancing rapidly and are expected to enter clinical evaluation soon [60].

### 5.1. Modulating the Immune Response

Given the central role of inflammation, immunomodulation has long been an attractive strategy. However, clinical trials of broad anti-inflammatory agents have yielded disappointing or mixed results. Therapies targeting single cytokines, such as anti-TNF antibodies or IL-1 receptor antagonists, have largely failed to improve outcomes in unselected sepsis populations, likely due to the complexity and redundancy of the inflammatory network and the heterogeneity of patient responses [61,62,63]. The use of corticosteroids remains controversial, with potential benefits in certain patient subgroups but also risks of immunosuppression and secondary infections [64].

The future of immunotherapy in sepsis likely lies in a more personalized approach. The identification of distinct immune phenotypes, such as the hyper-inflammatory macrophage activation syndrome (MAS)-like phenotype associated with coagulopathy and liver dysfunction, offers an opportunity to stratify patients for targeted therapies [31]. Guiding treatment with real-time immune profiling and biomarkers may allow for the application of potent immunomodulatory drugs to the right patient at the right time, avoiding the pitfalls of a “one-size-fits-all” approach.

### 5.2. Targeting Metabolic and Mitochondrial Pathways

As the importance of metabolic failure has become clearer, therapies aimed at restoring hepatic metabolism and protecting mitochondria have emerged as a promising frontier.

Preclinical studies have shown that activating PPARα with agonists like pemafibrate can restore fatty acid oxidation and improve outcomes. Dichloroacetate, an inhibitor of pyruvate dehydrogenase kinase, has been shown to reverse sepsis-induced hepatic metabolic dysfunction in animal models by promoting glucose oxidation and improving systemic energy balance. Notably, pemafibrate, a drug originally developed for treating dyslipidemia, exemplifies how existing compounds can be repurposed for new therapeutic applications. With regulatory pathways increasingly recognizing the potential of repurposed drugs, there is an opportunity to expedite clinical trials. For instance, medications targeting metabolic pathways often benefit from accelerated approval programs, which have facilitated the rapid transition from promising preclinical results to clinical testing. This encourages confidence in the feasibility and rapid translation of metabolic modulators, such as pemafibrate, into therapies for sepsis-associated liver injury.

Given the role of oxidative stress, antioxidants are a logical intervention. However, general antioxidants have had limited success. A more targeted approach focuses on protecting mitochondria directly. Mitochondria-specific scavengers, such as Mito-TEMPO, have demonstrated efficacy in preclinical models by reducing mitochondrial ROS, attenuating hepatocyte pyroptosis, and mitigating liver injury [40,65,66].

### 5.3. Restoring the Gut–Liver Axis

Targeting the gut to protect the liver is a rapidly evolving and highly promising therapeutic strategy. The goal is to restore gut microbiota homeostasis, strengthen the intestinal barrier, and reduce the translocation of inflammatory stimuli. Several approaches are under investigation:

Probiotics, prebiotics, and synbiotics aim to replenish beneficial bacteria and restore a healthy microbial balance. Fecal microbiota transplantation (FMT), the transfer of a healthy donor’s stool, has shown remarkable efficacy in other dysbiosis-related conditions and is being explored for sepsis [45].

Restoring protective microbial metabolites is another avenue. Supplementation with indole-3-propionic acid (IPA) has been shown to alleviate SALI in animal models by activating the PXR pathway and reducing inflammation [24].

Drugs like metformin have been shown to modulate the gut microbiota and improve intestinal barrier function, offering a potential repurposing opportunity for sepsis [61,67].

While these strategies are highly encouraging in preclinical settings, robust clinical trials are urgently needed to validate their efficacy and safety in human sepsis. Notably, several clinical trials are either ongoing or planned to further explore these therapies. For example, trials assessing the efficacy of dichloroacetate, mitochondrial antioxidants, and fecal microbiota transplantation in sepsis-associated liver injury are already underway. Such studies are crucial for translating these promising interventions from the laboratory to clinical practice and present potential opportunities for collaboration among researchers in the field [7,13].

### 5.4. Emerging and Novel Strategies

Research continues to uncover novel molecular targets. For instance, protein kinase C-alpha (PKCα) has been identified as a key mediator of liver injury, and targeting it with inhibitors has been shown to prolong survival and restore liver function in preclinical sepsis models [68]. Compounds derived from traditional Chinese medicine are also being investigated for their multi-target anti-inflammatory and antioxidant properties [69,70]. Finally, advances in nanotechnology are enabling the development of drug delivery nanosystems designed to specifically target injured liver cells, which could enhance the efficacy of therapeutic agents while minimizing systemic side effects [71].

## 6. Gaps in Knowledge and Future Research Directions

Despite significant progress, critical gaps in our knowledge remain, hindering the translation of basic science discoveries into effective clinical practice. Future research must be strategically directed to bridge these divides.

The most significant gap is the poor translation of findings from animal models to human patients. Rodent models of sepsis, such as cecal ligation and puncture (CLP) or LPS injection, have been invaluable for dissecting molecular pathways but often fail to recapitulate the heterogeneity and complexity of human sepsis [13,72,73]. Some specific limitations include the inability of these models to fully mimic human immune responses, including the diversity of immune cell types and their interactions, as well as the differences in hepatic microcirculatory disturbances observed in humans. Additionally, the temporal progression of organ failure and recovery is poorly represented in small-animal models. Future research must prioritize the development and use of more clinically relevant models, including large-animal models (e.g., porcine) and advanced in vitro systems such as human liver organoids and multi-organ-on-a-chip platforms, which better mimic human liver responses [32,74]. Ultimately, well-designed, prospective clinical trials are indispensable for validating promising therapeutic targets.

While the importance of the gut–liver axis is established, many mechanistic details remain obscure. A key priority is to move beyond association to establish causality. This includes identifying the specific microbial species and consortia responsible for producing protective metabolites, such as IPA, and understanding how their abundance changes during sepsis [24]. Validating microbiota-targeted therapies (e.g., probiotics, FMT) through rigorous, standardized randomized controlled trials is a high-priority area to move these promising interventions into clinical practice [7,16].

Sepsis is not a monolithic disease. Patient heterogeneity in terms of genetics, comorbidities, and immune status is a major reason for the failure of past clinical trials. Future research should focus on developing and validating biomarker panels to stratify patients into distinct endotypes (e.g., hyperinflammatory vs. immunosuppressed). This would enable personalized immunotherapy, directing potent drugs to patients most likely to benefit while sparing others from potential harm [31,61,75].

Research has often studied inflammatory and metabolic pathways in isolation. A systems biology approach employing multi-omics technologies (genomics, transcriptomics, proteomics, metabolomics) is needed to create integrated maps of the crosstalk among these pathways. This will provide a more holistic understanding of SALI and may reveal novel therapeutic targets at the intersection of metabolism and inflammation [15,37].

The lack of reliable early biomarkers remains a major clinical obstacle. High-priority research should focus on the large-scale clinical validation of the most promising novel biomarkers, including ncRNAs, protein panels, and dynamic liver function tests. The goal is to develop a standardized, accessible diagnostic toolkit that facilitates early diagnosis, guides therapeutic decisions, and provides accurate prognostication [54,76].

A structured validation roadmap can help to guide this research agenda. The discovery phase should prioritize identifying potential biomarker candidates through extensive screening and initial validation. This phase must be followed by verification efforts that reproduce findings across different lab settings, ensuring robustness and reliability. Finally, a multicenter trial phase is critical, involving standardized protocols across diverse populations and healthcare settings to evaluate the efficacy and generalizability of these biomarkers in clinical practice. Establishing this clear roadmap can accelerate the journey from research to clinical implementation, turning novel biomarkers from promising concepts into integral parts of clinical decision-making.

## 7. Conclusions

Sepsis-associated liver injury is a serious condition caused by a mix of immune problems, metabolic failure, and disruption of the gut–liver axis. Research shows there are many causes. Early increases in proinflammatory cytokines, activation of pathways such as NF-κB and the NLRP3 inflammasome and resulting oxidative stress directly harm liver cells. This is made worse by a severe metabolic crisis in the liver, including mitochondrial failure, low energy production, and issues with regulators like PPARα, which leads to fat buildup and further damage.

The gut–liver axis is increasingly recognized as a critical factor in SALI, as gut dysbiosis and compromised intestinal barrier function permit greater translocation of microbial products to the liver, thereby exacerbating inflammation. Despite advances in understanding driven by novel research methodologies, a substantial gap persists between mechanistic insights and the development of effective therapies. Future research priorities include establishing more representative experimental models, conducting robust clinical trials for emerging therapies, and implementing personalized medicine approaches that utilize patient-specific biomarkers and immune profiles. The overarching objective is to progress beyond supportive care by developing interventions that modulate inflammation, restore metabolic function, and repair the gut–liver axis, ultimately improving patient recovery.

## Figures and Tables

**Table 1:** The Pathophysiological Triumvirate of Sepsis-Associated Liver Injury (SALI).

Mechanism	Key Features	Main Drivers	Consequences
Immune Dysregulation/Inflammatory Cascade	Early “cytokine storm” with TNF-α, IL-6; activation of NF-κB&NLRP3 inflammasome	Kupffer cell overactivation, neutrophil recruitment & NET formation, complement activation	Hepatocellular damage, ROS release, pyroptosis, later immunosuppression ("immunoparalysis")
Metabolic Reprogramming and Mitochondrial Dysfunction	Shift from anabolic to catabolic metabolism; impaired fatty acid oxidation (FAO)	Downregulation of PPARα & HNF4α; oxidative stress damaging mitochondria	Energy crisis (↓ATP), lipid accumulation (lipotoxicity), failed autophagy/mitophagy→ apoptosis/pyroptosis
Gut–Liver Axis Disruption	Gut dysbiosis & “leaky gut” microbial product translocation (e.g., LPS)	Loss of protective metabolites (e.g., IPA), gut-derived norepinephrine	Sustained hepatic inflammation, second-hit injury
Microcirculation and Transport Dysfunction	Sinusoidal endothelial injury, microvascular thrombosis	Neutrophil & platelet aggregation, PI3Kγ activation	Intrahepatic cholestasis (↓Mrp2), toxic bile acid buildup

**Table 2:** Clinical Manifestations and Diagnostic Challenges.

Feature	Details	Limitations
Common Presentation	Septic cholestasis: bilirubin, mild↑AST/ALT	Often late sign; may be asymptomatic early
Standard LFTs	ALT, AST, bilirubin	Late indicators, lack specificity, poor correlation with functional impairment
Novel Biomarkers	Procalcitonin (injury mediator), CAAP48; protein panels (ARG1, MDH1, GSTα, 5-NT, SDH); ncRNAs	Need large-scale validation
Dynamic Liver Function Tests	LiMAx (^13^C-methacetinmetabolism), ICG-PDR (plasma clearance)	Outperform LFTs for prognosis; limited availability

**Table 3:** Current and Emerging Therapeutic Strategies.

Target Area	Strategy	Examples	Evidence
Immune Modulation	Cytokine blockade, corticosteroids, phenotype-based immunotherapy	Anti-TNF, IL-1RA, immune profiling for MAS-like phenotype	Mixed/negative results in broad trials; future lies in personalized approach
Metabolic & Mitochondrial Support	Restore FAO, protect mitochondria	PPARα agonists (pemafibrate), dichloroacetate. Mito-TEMPO	Effective in preclinical models; human data lacking
Gut-Liver Axis Restoration	Microbiota modulation, metabolite supplementation, barrier protection	Probiotics, prebiotics, synbiotics, FMT, IPA supplementation, metformin	Promising animal data; trials needed
Novel Molecular Targets	Block PKCα, multi-target natural compounds, nanomedicine delivery	PKCα inhibitors, traditional Chinese medicine extracts, targeted nanosystems	Preclinical stage

## Data Availability

No new data were created or analyzed in this study.
